# Early Form-Based Morphological Decomposition in Tagalog: MEG Evidence from Reduplication, Infixation, and Circumfixation

**DOI:** 10.1162/nol_a_00062

**Published:** 2022-02-16

**Authors:** Samantha Wray, Linnaea Stockall, Alec Marantz

**Affiliations:** Neuroscience of Language Lab, New York University Abu Dhabi, Abu Dhabi, UAE; Department of Linguistics, Queen Mary University of London, London, UK; Linguistics and Psychology, New York University, New York, USA; Department of Linguistics, Dartmouth College, Hanover, New Hampshire, USA

**Keywords:** magnetoencephalography, morphology, visual word recognition, Tagalog, M170, reduplication

## Abstract

Neuro- and psycholinguistic experimentation supports the early decomposition of morphologically complex words within the ventral processing stream, which MEG has localized to the M170 response in the (left) visual word form area (VWFA). Decomposition into an exhaustive parse of visual morpheme forms extends beyond words like *farmer* to those imitating complexity (e.g., *brother*; Lewis et al., 2011), and to “unique” stems occurring in only one word but following the syntax and semantics of their affix (e.g., *vulnerable*; Gwilliams & Marantz, 2018). Evidence comes primarily from suffixation; other morphological processes have been under-investigated. This study explores circumfixation, infixation, and reduplication in Tagalog. In addition to investigating whether these are parsed like suffixation, we address an outstanding question concerning semantically empty morphemes. Some words in Tagalog resemble English *winter* as decomposition is not supported (*wint*-*er*); these apparently reduplicated pseudoreduplicates lack the syntactic and semantic features of reduplicated forms. However, unlike *winter*, these words exhibit phonological behavior predicted only if they involve a reduplicating morpheme. If these are decomposed, this provides evidence that words are analyzed as complex, like English *vulnerable*, when the grammar demands it. In a lexical decision task with MEG, we find that VWFA activity correlates with stem:word transition probability for circumfixed, infixed, and reduplicated words. Furthermore, a Bayesian analysis suggests that pseudoreduplicates with reduplicate-like phonology are also decomposed; other pseudoreduplicates are not. These findings are consistent with an interpretation that decomposition is modulated by phonology in addition to syntax and semantics.

## INTRODUCTION

The process of word recognition is necessarily complicated for words composed of multiple morphemic constituents. Are morphologically complex words decomposed during lexical access? Does this decomposition occur early in the word recognition pipeline before meaning is associated with morphemic units, and what aspects of a word’s internal structure determines this? The current study aims to contribute unstudied morphological phenomena to the growing body of literature focused on early form-based morphemic decomposition.

### Visual Word Recognition

Full decomposition models (contra non-decompositional models, i.e., Giraudo & Grainger, 2000) posit an early automatic form-based decomposition of complex words into the orthographic forms of their constituent morphemes during visual lexical access (including Crepaldi et al., 2010; Taft, 1979, 2004; Taft & Forster, 1975).

Much evidence delineating the discriminatory nature of this morphological parser has emerged. In masked priming studies, *teacher* primes TEACH but *brother* also primes BROTH, despite the fact that the orthographic -*er* is not an affix in that word (Rastle & Davis, 2008; Rastle et al., 2004). This contrasts with the lack of priming between *brothel* and BROTH (Rastle et al., 2004), where -*el* is not a visual form of an English morpheme. Neural evidence from magnetic resonance imaging (MRI; Gold & Rastle, 2007), magnetoencephalography (MEG; Cavalli et al., 2016; Fruchter & Marantz, 2015; Lehtonen et al., 2011; Lewis et al., 2011), and electroencephalography (EEG; Beyersmann et al., 2014; Lavric et al., 2007; Morris & Stockall, 2012; Morris et al., 2007, 2008; Royle et al., 2010) further support a semantics-independent morphological parser as the responsible mechanism for this phenomenon. MEG research by Tarkiainen et al. (1999), and fMRI studies by Dehaene et al. (2002) localized a possible neural basis for character string processing to the fusiform gyrus, specifically the visual word form area (VWFA). In MEG, this region has been shown to be a generator of a visually evoked response component peaking approximately 170 ms after stimulus onset (the M170) that was originally targeted for possible relevance for morphology as a bilateral component sensitive to a word’s exhaustive parsability (Zweig & Pylkkänen, 2009). In subsequent studies, the left M170 was found to index several lexical variables associated with morphological parsing, including affix frequency and the transition probability from a stem to the whole word, both for bound stems and free stems (Solomyak & Marantz, 2010). The ERP analog to the M170 response appears to be the N250, which consistently shows effects of morphological priming but not semantic priming in the studies cited above (see Morris & Stockall, 2012, and Royle & Steinhauer, in press, for reviews and discussion of this literature).

M170 activity elicited by *brother* words correlates with the stem:whole word transition probability (often abbreviated as TP or TPL in the literature) given a stem of *broth*, just as the M170 evoked by genuinely complex words like *teacher* correlates with the stem:whole word transition probability given the stem *teach*; this is not true for *brothel* words (Lewis et al., 2011). In addition to this dependence of decomposition on the presence of an affix, a viable stem must result from the parse stripping the suffix, as evidenced by the comparison between *brother* and *winter* (Zweig & Pylkkänen, 2009), where *winter* patterns with the morphologically simple words given the non-existence of a stem *wint*. The stem involved in an exhaustive morphological parse may be bound, provided the word follows morphosyntactic rules associated with its suffix. Thus, M170 activity is predicted by a model computing the M170 from transition probability (and other variables) for *vulnerable* (from the unique bound stem *vulner* to the suffix -*able*, a transition probability of 1) as it is morphosyntactically and semantically congruent with other adjectives with the -*able* affix. This is not the case for e.g., *sausage* (from *saus* to *age*, also a transition probability of 1), since the combination of *saus*(*e*) and *age* would not conform to any rule in English, given the meaning of *sausage* (Gwilliams & Marantz, 2018).

A summary of the previous results in the literature on morphological processing in occipito-temporal regions is presented in Table 1.

**Table 1.** A summary of MEG studies demonstrating the correlation of morphological variables, including transition probability (TP), with activity in occipito-temporal regions

**Study**	**Morphological variable**	**Timing and laterality**	**Morphological type**	**Language**
Zweig and Pylkkänen (2009)	complexity	prefix: 174–182 ms bilateral	prefixation, suffixation	English
		suffix: 170–186 ms right hemisphere		
Vartiainen et al. (2009)	complexity	200–800 ms left hemisphere (temporal)	suffixation	Finnish
Solomyak and Marantz (2010)	stem:whole word TP	178–214 ms left hemisphere	suffixation	English
Lewis et al. (2011)	stem:whole word TP	164–208 ms left hemisphere	suffixation	English
Lehtonen et al. (2011)	stem:suffix TP for low semantic opacity	220 ms left hemisphere	suffixation	English
Fruchter et al. (2013)	morphophonological congruency	158–183 ms left hemisphere	irregular	English
Gwilliams and Marantz (2018)	stem:whole word TP	150–180 ms left hemisphere	suffixation	English
Neophytou et al. (2018)	stem:whole word TP	100–200 ms left hemisphere	suffixation	Greek
Hakala et al. (2018)	Morfessor (minimum description length) (Creutz & Lagus, 2007; Rissanen, 1978)	140–200 ms bilateral	suffixation	Finnish
Ohta et al. (2019)	root:affix TP	150–200 ms left hemisphere	suffixation	Japanese
Stockall et al. (2019)	stem:whole word TP	200–220 ms right hemisphere	prefixation	English

The current study expands upon these studies typologically, and more generally informs our knowledge of automatic decomposition during early visual word recognition. The study allows us to determine if previously attested automatic decomposition effects and their accompanying theories extend from languages with relatively more simplistic morphological processes to those with more complicated processes. Moreover, Tagalog exhibits morphologically triggered phonological phenomena that allow us to determine whether phonological cues to morphological complexity are attended to in early visual processing. The results of the current study are consistent with those in Table 1 that demonstrate the correlation of M170 activity with morphological measures, suggesting that the effects of a complex word’s internal structure modulate activity in anterior fusiform gyrus regardless of the morphological process underlying that word’s complexity. Support for this conclusion is composed of results from seven word types: (i) reduplicated words; (ii) pseudoreduplicated words that exhibit phonological behavior indicative of morphological complexity; (iii) pseudoreduplicated words that do not exhibit phonological behavior indicative of morphological complexity; (iv) infixed words; (v) non-infixed words with a phono-orthographic string that could be an infix (i.e., a *winter* type); (vi) circumfixed words; and (vii) unambiguously morphologically simple words not imitative of complexity. Relevant morphophonological details are reviewed in the sections that immediately follow.

### Reduplication in Tagalog

The current study includes a focus on phonological transparency as a perceptual cue to morphological complexity.

Reduplication in Tagalog can feed transparently applied phonological rules, creating phonological non-identity between the base and copy (reduplicant). (We use the term *rule* to refer to emergence of phonological phenomenon. Whether this occurs in a serial application, or as Zuraw, 2002, suggests, via the ranking of Optimality Theoretic constraints, is beyond the scope of the current study and has no bearing on the results discussed within.) However, reduplicates in Tagalog can also exhibit a non-transparent application of phonological rules, keeping base and copy more similar phonologically than they would be if the rules applied normally. In non-transparent application, phonological rules apply to both the base and the reduplicant despite the fact that only one of the segments fulfills the environmental requirements for application of the rule, or fail to apply even though one of the segments falls into the usual triggering environment (Carrier, 1979; Marantz, 1982; McCarthy & Prince, 1995; Wilbur, 1973). An example of failure to apply a rule governing the raising of the vowel /o/ to /u/ in reduplication is shown in (1b). Contrast this with transparent application in suffixation in (1a).

(1)  Phonological rule application and suffixation/reduplication
     Stem            Complex form
     a. tap**o**s   “ending”     tap**u**sin    “to be finished” (Zuraw, 2009)
     b. b**o**to    “vote”     b**o**b**o**to    “will vote”

### Pseudoreduplication in Tagalog

There is a class of Tagalog words that superficially appear to be reduplicated but do not have an independent stem and lack the morphosyntax of a reduplicated word (termed “pseudoreduplicates” by Zuraw, 2002). Attempts to reduce the repeated orthophonological material to a base and reduplicating morpheme both violate stem minimality constraints in Tagalog (stems are generally bi-syllabic) and are rejected by native speakers as words of the language. Examples of pseudoreduplicates are shown in (2).

(2)  Pseudoreduplicated words (Zuraw, 2002)
     a. mismis    “scraps”     *mis
     b. luloŋ    “swallowing”    *loŋ
     c. ŋasŋas    “scandal”     *ŋas

For a subset of these pseudoreduplicated words, phonological rules are applied transparently with no exceptions for identity between the *base* and *reduplicant*, consistent with the word being morphologically simple. For a minority of the pseudoreduplicated words, however, a rule is over-/underapplied, much as it would be for a true reduplicated word. Examples of pseudoreduplicants exhibiting transparent and non-transparent application are shown in (3). Pseudoreduplicated words which exhibit non-transparent application of phonological rules are marked with [+i] as they phonologically *imitate* true reduplicates; those which transparently apply phonological rules as expected of morphologically simple words are marked with [−i].

(3)  Transparent and non-transparent phonology in pseudoreduplicates (Zuraw, 2002)
     a. dubdob   “vehemence”  Transparent application [−i]
     b. gonggong  “grunt fish”   Non-transparent application [+i]

Native speaker judgment for items in the current study placed a certain degree of variability on non-transparent application of the vowel height rule for pseudoreduplicated words, in addition to the variability noted by Zuraw (2002). If the underapplication of the vowel height rule was acceptable, the word was considered to have non-transparent application, even if the transparent form was also considered acceptable.

The current study aimed to answer the question: Are [−i] pseudoreduplicated words that transparently apply rules processed differently than those [+i] pseudoreduplicated words that do not? Specifically, given that non-transparent application makes a pseudoreduplicated word appear more like a product of morphological reduplication, are these [+i] pseudoreduplicated words processed like reduplicated words? If pseudoreduplicated words are decomposed in parallel to truly reduplicated words, the neurolinguistic evidence would support Zuraw’s (2002) hypothesis that these words are represented with a syntactically and semantically null reduplicating morpheme.

### Infixation in Tagalog

In Tagalog, an infix follows the first consonant of the base (Schachter & Otanes, 1983). Tagalog utilizes several infixes, including -*in*- which marks patient focus. Examples of this infix are shown in (4).

(4)  -*in*- Infixation
         Stem            Infixed
     a.   subok    “try”       sinubok    “tried”
     b.   gapos    “cord”      ginapos    “tied/banned”
     c.   gulat    “surprise”     ginulat    “shocked someone”

Tagalog also has words with initial syllables ending in /in/ which are not morphologically complex. In this way, these words are analogous to previously studied word types in English discussed in detail above that contain phono-orthographic strings consistent with an affix but that are not treated as morphologically complex by visual perception areas in the brain sensitive to relations between morphemes. Specifically, much like *winter* or *sausage*, the stripping of the affix does not result in a viable stem, and furthermore the word is not morphosyntactically congruent with words that contain the affix (Gwilliams & Marantz, 2018; Zweig & Pylkkänen, 2009). Examples of words with initial syllables ending in /in/ that are morphologically simple appear in (5). Note that there is no isolable stem in these words, and they do not exhibit the morphosyntax indicative of -*in*- infixed words (namely, the words are not perfective verbs). We term these words *pseudo-infixed*.

(5)  Pseudo-infixed /in/
     a. ministro    “ministry”    *mistro
     b. ninoŋ     “godfather”   *noŋ
     c. pinsaŋ     “cousin”    *pisaŋ

The current study then aims to discover if pseudo-infixed words are processed as the evidence from English processing predicts (i.e., *broth*-*er* vs. *winter* (Zweig & Pylkkänen, 2009); *excurs*-*ion* vs. *sausage* (Gwilliams & Marantz, 2018)). If morphosyntactic indexing and stem viability are coded for Tagalog infixes in much the same way as they are for English suffixes, we expect that the pseudo-infixes will not be automatically stripped during the word recognition process.

### Predictions and Design

The present study aims to explore the implications of Tagalog morphology, including reduplication, infixation, and circumfixation, for the early evoked activity in occipito-temporal cortex associated with automatic decomposition in visual word recognition models. Furthermore, the study aims to determine whether words that appear to be reduplicated or infixed based on their written form are automatically decomposed, and what modulates this decomposition. The study includes two blocks, run in the same experimental session. Block 1 investigates processing of words formed through reduplication and words with circumfixes. Block 1 also compares real reduplicated words to [−i] pseudoreduplicated words which transparently apply phonological rules and [+i] pseudoreduplicated words which non-transparently apply rules (i.e., are reduplicate-like). Block 2 compares processing of infixed words to pseudo-infixed words that superficially appear to have an infix but that are morphologically simple.

A summary of the design of the two blocks with accompanying hypotheses about decomposition for each word type is presented in Table 2.

**Table 2.** Conditions of the MEG experiment investigating the processing of reduplicated and infixed forms, and words that orthographically appear to be reduplicated or infixed but are morphologically simple

**Condition**	**Sample item**	**Prediction for decomposition**	**Results for decomposition**
**Block 1**			
simple	aberya “flawed”	✗	✗
reduplicated	araw-araw “everyday”	✓	✓
[−i] pseudoreduplicated: transparent phonology	musmos “naïve”	✗	✗
[+i] pseudoreduplicated: non-transparent phonology	gonggong “grunt fish”	✓	✓
circumfixed	ka-ruwag-an “cowardice”	✓	✓
**Block 2**			
simple	lungkot “sadness”	✗	✗
infixed -*in*-	t-in-awag “called”	✓	✓
pseudo-infixed /in/	bintang “accusation”	✗	✓
circumfixed	ka-bayar-an “payment”	✓	✓

*Note*. The simple condition contains unambiguously simple words that have no orthographic imitation of complexity. Hyphens are included within words to indicate morpheme boundaries.

Note that there is an inconsistent distribution of parts of speech across conditions, as words which have reduplication or circumfixation as their only means of varying morphological complexity tend to be nouns, whereas infixed words tend to be verbs. However, transition probability is the feature of interest, and it has been demonstrated to influence the processing of both nouns and verbs, even within the same experiment (Lewis et al., 2011).

This experiment tests several hypotheses about what information is used in early, automatic morpheme segmentation by the visual system, and from which morphemes this information is accessible. First, we address the hypothesis that circumfixed, infixed, and reduplicated words will be processed as a function of their morphemic transition probability, as has been attested for English, Greek, and Finnish suffixes. Under this hypothesis, pseudo-infixed words will not be automatically parsed. Furthermore, we hypothesize that the decomposition of pseudoreduplicated words will be modulated by phonological transparency, as those that imitate reduplicated words by virtue of their nontransparent application of phonological rules will be processed as if they are reduplicated.

## MATERIALS AND METHODS

### Participants

Twenty right-handed participants took part in the study (13 females, ages 24–46, mean age = 33). A language history was collected, and speakers who self-reported being native speakers of Tagalog were retained in the study; speakers who self-reported their native language as another Filipino language such as Cebuano/Bisaya were not retained. All participants reported normal or corrected-to-normal vision. Written informed consent was obtained from all individuals prior to participation in the experiment.

### Materials

Stimuli were selected from a Tagalog dictionary (English, 1965), in addition to words identified by Zuraw (2002). Frequency counts were taken from a 5-million-word Wikipedia corpus (Oco & Roxas, 2012). Finally, the stimuli were vetted by a native speaker for lexicality and decomposability (defined as the ability to isolate a definable stem). To determine whether or not each word transparently applied phonological rules, the native speaker also provided judgments on forms that incorporated additional affixation not utilized in the experiment. A summary of the properties of the stimuli is presented in Table 3.

**Table 3.** Properties of items included as visual lexical decision stimuli in experiments with concurrent MEG

**Condition**	**Average frequency in parts per million (*SD*)**	**Average length in letters (*SD*)**
**Block 1**		
reduplicated	1.11 (±0.85)	7.5 (±1.46)
pseudoreduplicated: transparent application	1.19 (±1.17)	5.4 (±0.61)
pseudoreduplicated: non-transparent application	1.03 (±2.51)	6.3 (±0.87)
circumfixed	1.06 (±0.76)	9.5 (±0.97)
**Block 2**		
infixed -*in*-	18.9 (±26.22)	7.4 (±1.07)
pseudo-infixed /in/	21.1 (±29.47)	6.5 (±1.54)
circumfixed	17.4 (±24.13)	9.1 (±0.96)

Nonwords in both blocks were created using the nonce word generator toolkit Wuggy (https://crr.ugent.be/programs-data/wuggy; Keuleers & Brysbaert, 2010) by scrambling possible syllables using real Tagalog words as training input (). Then, an appropriate number of the nonce stems underwent the morphological processes in Table 3. For example, an equal number of nonce stems was “reduplicated” to the reduplicated items included as target items in the experiment. This was simply to ensure that participants did not develop a strategy for decisions that obscured the desired results.

Although circumfixed items were consistently the longest items in length of letters, and frequency was only matched within block and not across blocks, both length and frequency were added as fixed effects in the linear mixed effects model (described in detail in the Analysis section) so that they did not confound an analysis focusing on condition.

### Procedure

Data were collected at New York University Abu Dhabi overseen by New York University Abu Dhabi’s Institutional Review Board. Before beginning, all participants provided informed, written consent. Participants lay supine in a dimly-lit magnetically shielded room while stimuli were presented on a screen suspended 85 cm above the head. Stimuli were presented in black Times New Roman font (corresponding to a display size of 2 cm) against a grey background using the experiment control software Presentation (https://www.neurobs.com/; Neurobehavioral Systems). Pre-stimulus presentation of a fixation cross in the middle of the screen lasted for 50 ms. Stimulus order was fully randomized across and between 5 sets for each block, and participants were directed to indicate via button press with the non-dominant (left) hand whether they recognized each word as a word of their language or not. Participants were instructed to answer as quickly and as accurately as possible. After each set, participants could take a self-timed break during which they could perform small movements to remain comfortable. A short break also occurred between blocks 1 and 2. The total time for the experiment averaged 20 min.

MEG data were continuously recorded concurrently with accuracy and reaction time (RT) data. MEG data were recorded with a 1000 Hz sample rate on a 208-channel axial gradiometer system (Kanazawa Institute of Technology, Kanazawa, Japan) and went through an online low-pass filter at 200 Hz and a high-pass filter at 0.1 Hz.

Participants’ head shapes were digitized for source localization and coregistration using a FastSCAN laser scanner (Polhemus, VT, USA). Digitized head shapes were downsampled to create a smoothed surface using the FastSCAN software. Digital fiducial points were marked for each participant across the forehead, the anterior of the left auditory canal, and the anterior of the right auditory canal. Marker coils were taped to each participant’s head where the fiducials were recorded. A measurement of marker coil position was taken before and after each block to correct for participant movement post-hoc.

### Analysis

The first step in preprocessing MEG data was noise removal from the raw data using eight reference channels located away from the individual’s head and using the continuously adjusted least squares method (Adachi et al., 2001), which was performed using the MEG160 software (Yokohawa Electric Corporation and Eagle Technology Corporation, Tokyo, Japan). Subsequent preprocessing and analysis of MEG data were performed using MNE-Python (Gramfort et al., 2013, 2014) and Eelbrain 0.25.2 (Brodbeck, 2017) an independent components analysis (ICA, specifically FastICA) was performed on the full noise-reduced data to isolate and remove components corresponding to biomagnetic artifacts, such as eye movement (blinks, saccades) and pulse. Following ICA, the data went through a low-pass infinite impulse response 4th order Butterworth forward-backward filter with an upper cutoff frequency of 40 Hz. The data was epoched from 500 ms preceding stimulus onset to 500 ms following stimulus onset. Manual rejection of epochs to remove those contaminated by motor artifacts as well as those with activity exceeding +/−2,000 fT/cm was performed using Eelbrain, resulting in removal of 1.7% of trials. Epochs were not baseline corrected. Rather, 50 ms preceding the fixation cross were included as a fixed effect in the linear mixed effects model, following Alday (2019).

MEG data were coregistered with the FreeSurfer average brain (CorTechs Labs Inc., La Jolla, CA, USA) by manually scaling the participants’ digitized head shapes and the FreeSurfer average skull. An ico-4 source space was created consisting of 5,124 sources using a cortically constrained minimum norm estimate model (Hämäläinen & Ilmoniemi, 1994). Signed minimum estimates were used based on previous research showing their superiority to unsigned estimates in studying orthographic processing (Gwilliams et al., 2016). For each source, a boundary element model (Mosher et al., 1999) was used to compute the forward solution. The inverse solution using the forward solution was calculated and subsequently applied to the data with a fixed orientation of the dipole current. A signed fixed orientation for the source estimates was used to calculate the inverse solution, such that the direction of the current was defined and dipoles were perpendicular to the cortical surface. Finally, the data were noise-normalized in the spatial dimension, resulting in a dynamic statistical parameter map (dSPM; Dale et al., 2000).

Using the anterior fusiform functional region of interest (fROI) defined by Gwilliams et al. (2016), activity averaged across space was plotted using MNE-Python (Gramfort et al., 2013, 2014) for the M170 to be manually identified. Further analyses on this data were performed by using activity averaged across space and time as input for linear mixed effects models using R 3.6.1 (R Core Team, 2019) and lme4 (v1.1-21; Bates, Maechler, et al., 2015).

Behavioral data (specifically, RTs and accuracy) were analyzed using linear mixed effects models also using R (R Core Team, 2019) and lme4 (Bates, Maechler, et al., 2015). Items below chance accuracy were excluded from all analyses except the analysis of accuracy.

## RESULTS

### MEG Data

#### Complex words

**Figure 1.**
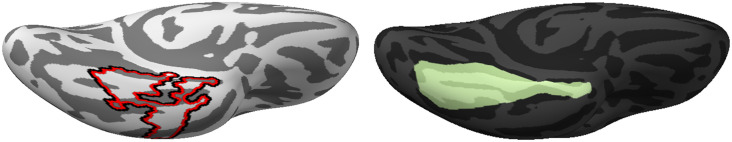
Ventral view of region of interest (ROI) for M170: VWFA (left) using coordinates from Gwilliams et al. (2016), located approximately in anterior fusiform gyrus (right). Shows inflated cortical surface of FreeSurfer average subject (Fischl, 2012). Plot was created in MNE-Python (Gramfort et al., 2013, 2014).

**Figure 2.**
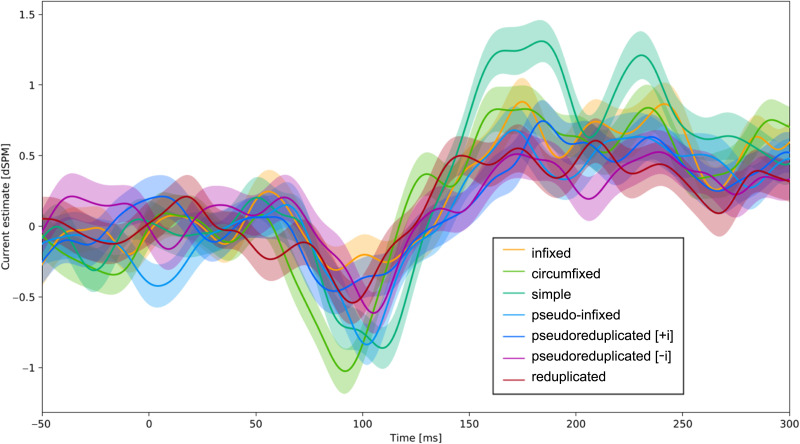
Time course and average activity (current estimates in unitless *z*) in VWFA from time of stimulus presentation to 300 ms after stimulus presentation. Shaded areas represent standard error of the mean. Plot was created in Eelbrain (Brodbeck, 2017).

Analyses were focused on activity in the left hemisphere fusiform gyrus (Figure 1), specifically in the anterior region identified by Gwilliams et al. (2016) as an fROI, plotted in Figure 1. Gwilliams et al. (2016) identified this fROI by running an English adaptation of the Tarkiainen et al. (1999) study on “Type Two” responses associated with the perception of visible letter strings vs. those obscured with visual noise, which was earlier and more posterior, and the perception of letter strings vs. symbol strings, which was later and more anterior. Crucially, they demonstrated that activity in the anterior region correlated with transition probability from morphologically complex English words (Solomyak & Marantz, 2010), and were able to spatiotemporally separate this response from activity associated with the visual noise manipulation. We selected 150–200 ms as the time window for analysis and the most likely candidate for the M170. As presented in detail in the Visual Word Recognition section, previous research has variously identified time windows of 100–200 ms (Fruchter et al., 2013; Neophytou et al., 2018; Stockall et al., 2019), 130–180 ms (Gwilliams et al., 2016), 150–180 ms (Gwilliams & Marantz, 2018), and 140–220 ms (Lewis et al., 2011). This selection appeared consistent with the wave form morphology; averaged activity from this fROI plotted by condition is shown in Figure 2.

Analysis of the neural results was completed in two steps. First, a linear mixed effects regression (LMER) was fit for activity elicited across all word types. Then, activity for simple words that could potentially be parsed as complex ([−i] pseudoreduplicated, [+i] pseudoreduplicated, pseudo-infixed) were compared to their truly complex counterparts using Bayesian estimation and evaluating the resulting posterior probability distributions.

For the first analysis, we used an LMER to investigate the effects of morphemic transition probability, as well as additional lexical properties, on left hemisphere dSPM averaged across space (the VWFA) as well as averaged across time (from 150 to 200 ms). Fixed effects in the model included the *base dSPM* of 50 ms pre-stimulus period (following Alday, 2019) with 50 ms selected as the pre-stimulus baseline time period to mirror the 50 ms time period of interest for post-stimulus dSPM, stem:whole word *transition probability*, word *length* in letters, natural log of stem *frequency* as continuous variables, as well as the fixed effect of the categorical variable *condition* (reduplicated, circumfixed, infixed -*in*-, simple, pseudo-infixed /in/, pseudoreduplicated [+i], pseudoreduplicated [−i]). The *interaction* of transition probability and condition was included in the model, and a by-subject intercept and by-subject slope of *length* were also included. The significance of fixed effects was determined using Wald tests on the coefficients using the Satterthwaite approximation for the degrees of freedom (implemented in the lmerTest package 3.1-1; Kuznetsova et al., 2017). Selection of the random effects proceeded via backward selection from the maximal model for both subject and item effects using the lmerTest package (Kuznetsova et al., 2017; for discussion, see Bates, Kliegl, et al., 2015; Barr, 2013; Barr et al., 2013; and Matuschek et al., 2017). Treatment coding was specified for condition, with the reference level being the reduplicated condition. To check for collinearity, the generalized variance inflation factor (GVIF) was calculated using the car package (Fox & Weisberg, 2019); when taking degrees of freedom into account, no GVIF was greater than 2.94. The full model summary after random effect reduction is shown in Table 4.

**Table 4.** Summary of the LMER showing correlation coefficients of lexical statistics and word types to source component amplitudes (*left* hemisphere)

**Formula:** dSPM ∼ base_dSPM + TP * condition + Length + BaseFreqlog + (1 | Subject) + (Length | Subject) + (BaseFreqlog|Subject)				
**Fixed effects:**	**Estimate**	***df***	***t* value**	**Pr(>|*t*|)**
(Intercept)	0.75	408.02	1.635	0.10286
Base dSPM	−0.14	4,333.88	−9.545	2e−16***
Transition Probability	0.56	4,295.51	1.371	0.17035
Condition = simple	0.35	4,295.31	0.975	0.32940
Condition = pseudo-infixed	0.78	4,295.53	−0.292	0.77000
Condition = pseudoredup [+i]	−0.56	4,295.31	−1.456	0.14560
Condition = pseudoredup [−i]	−0.79	4,295.48	−2.039	0.04146*
Condition = circumfix	0.73	4,294.6	2.776	0.00554**
Condition = infixed	0.78	4,294.64	2.397	0.01659*
Length	−0.04	213.07	−0.701	0.48392
log (Base Frequency)	−0.02	36.69	−0.469	0.64187
Interaction, TP:Condition = circumfix	−0.51	4,295.16	−0.949	0.34266
Interaction, TP:Condition = infixed	−1.20	4,295.12	−2.234	0.02554*
Signif. codes: 0 ‘***’ 0.001 ‘**’ 0.01 ‘*’ 0.05 ‘.’ 0.1 ‘ ’ 1				
**Random effects:**	**Variance**			
Subject	0.399313			
Length|Subject	0.009153			
Base Frequency|Subject	0.015711			
Residual	10.313368			

*Note*. Treatment coding was used for the categorical predictor condition, with the reduplicate condition serving as the reference level. Estimates have been rounded to 2 decimal places. Calculation of *p* values from *t* tests and *df*s were performed using Satterthwaite’s method in the lmerTest package (Kuznetsova et al., 2017).

**Figure 3.**
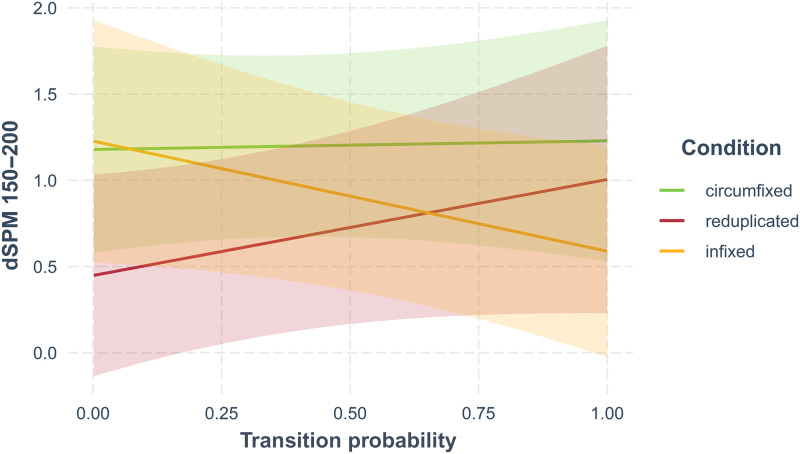
Average activity plotted against stem:whole word transition probability separated by word type. This illustrates an interaction between condition and transition probability. Shaded areas represent 95% confidence interval. Plot was created in R (R Core Team, 2019) using jtools 2.0.1 (Long, 2019).

There was a significant interaction between transition probability and the reduplicated and infixed levels of condition indicating that the effect of transition probability on dSPM was not consistent across morphological types. The effect of transition probability for reduplicated words was significantly different than for infixed words [*t*(4,295.12) = −2.23, *p* = 0.03]. There was no significant difference on the effect of transition probability for circumfixed words and reduplicated words [*t*(4,295.16) = −0.95, *p* = 0.34]. This is plotted in Figure 3, which shows that the relationship between transition probability and dSPM is positive for reduplicated and circumfixed words: As it becomes more likely for a whole word to contain its stem, more activity is elicited in the left hemisphere VWFA. This pattern is consistent with those attested for suffixation in English (Gwilliams & Marantz, 2018; Lewis et al., 2011; Solomyak & Marantz, 2010) and Greek (Neophytou et al., 2018). However, for infixed words, as it becomes more likely for a whole word to contain its stem, less activity is elicited. The morphologically simple words (conditions: simple, pseudo-infixed, pseudoreduplicated [+i], pseudoreduplicated [−i]) all have transition probabilities equal to 1, so there was no corresponding interaction term and the main effects can be interpreted directly. Of most interest are the comparisons between reduplicated and pseudoreduplicated [−i] as well as between reduplicated and pseudoreduplicated [+i]. There was a significant difference between reduplicated and pseudoreduplicated [−i] [*t*(4,295.48) = −2.039, *p* = 0.04]. This is consistent with the hypothesis that pseudoreduplicated [−i] would not be processed like reduplicated words, that is, they would not be automatically decomposed, because they are not phonologically imitative of reduplicated words. In contrast, there was no significant difference between reduplicated and pseudoreduplicated [+i] words [*t*(4,295.31) = −1.46, *p* = 0.15]. Finally, both length [*t*(213.07) = −0.70, *p* = 0.48] and stem frequency [*t*(36.69) = −0.47, *p* = 0.64] were not significant.

To determine if there was a bilateral effect, the process was repeated for the right-hemisphere homologue to the VWFA. No effect was found (see the results in the online Supporting Information; https://doi.org/10.1162/nol_a_00062).

#### Comparison between complex and pseudo-complex words

**Figure 4.**
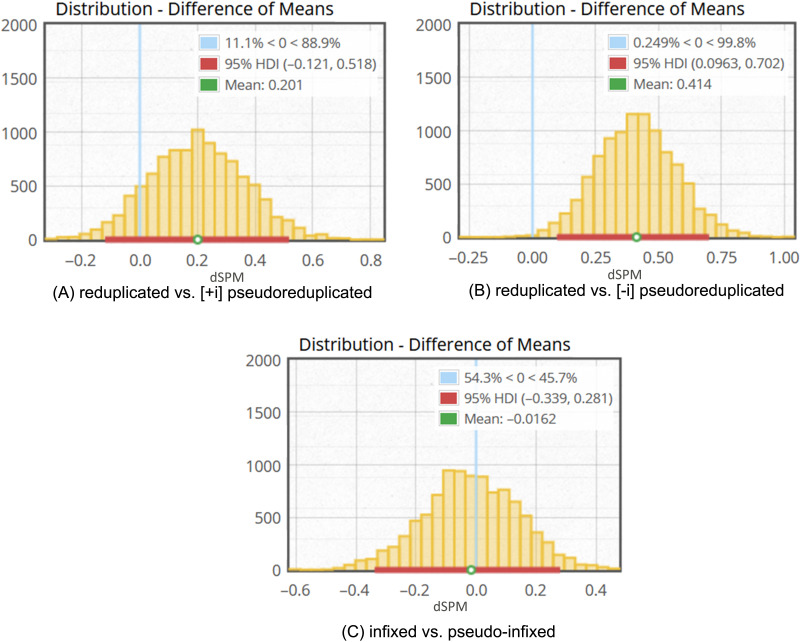
Histograms of differences of means produced by 10,000 MCMC samples per word type. The vertical light blue line marks 0 difference between the predicted means. The horizontal red line indicates the highest density interval (HDI), or 95% of the predicted difference of means. Plots are from the Bååth (2012) implementation of Kruschke (2013).

It is possible to evaluate comparisons between word types further by using a Bayesian parameter estimation approach. A posterior probability distribution was calculated for the difference in dSPM values between a complex word type (reduplicated and infixed) and its corresponding pseudo-word type ([+i] pseudoreduplicated, [−i] pseudoreduplicated, and pseudo-infixed), using Metropolis-within-Gibbs Markov chain Monte Carlo (MCMC) sampling with 10,000 samples (using the Bååth, 2012, implementation of Kruschke, 2013). Based on the posterior probability distribution, shown in the difference of means in Figure 4, we quantified the probability that word types elicited similar dSPM values based on comparing observed dSPM from complex and pseudo-complex types.

First, we begin with a comparison of reduplicated words and pseudoreduplicated words. Figure 4A and 4B demonstrates a contrast between pseudoreduplicated types. The difference between reduplicated and [+i] pseudoreduplicated, shown in 4A, is estimated to be credibly zero, as indicated by a 0 estimated difference of means being within the 95% highest posterior probability density interval. (An alternative approach is to specify a region of practical equivalence (for details see Kruschke, 2013) based on effect size and determine if 95% percent of the difference of means distribution falls within this.) This is indicative of equivalent values. This is consistent with an interpretation that [+i] pseudoreduplicated words and reduplicated words elicit similar dSPM values. In contrast, in Figure 4B, the difference between reduplicated words and [−i] pseudoreduplicated words was determined to be non-zero: a 0 estimated difference of means is outside the 95% likelihood density. This is consistent with an interpretation that [−i] pseudoreduplicated words and reduplicated words elicit different dSPM values.

Next, a comparison of infixed words and pseudo-infixed words was undertaken. This difference was also estimated to be credibly zero, as shown in Figure 4C. A 0 estimated difference of means is within 95% likelihood density.

Taken together, these provide evidence that [+i] pseudoreduplicated and pseudo-infixed words are processed like their complex (reduplicated) counterparts, whereas [−i] pseudoreduplicated transparent words are not. This is indicative of decomposition for two of the three pseudo-complex types. Our hypotheses stated that [+i] pseudoreduplicated nontransparent words would be automatically decomposed given that their phonology is imitative of reduplicated words, whereas [−i] pseudoreduplicated transparent words would not be.

### Behavioral Data

#### Reaction time

**Figure 5.**
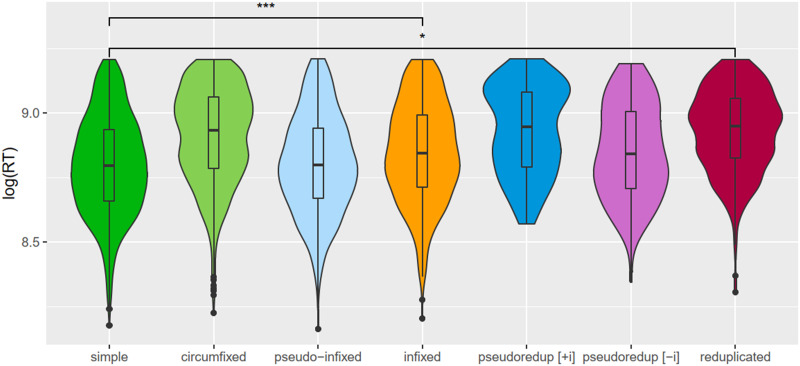
Violin plot showing a graphical summary of RTs. Comparisons between morphologically simple and other conditions are from the model in Table 5. Plot was created in R 3.6.1 (R Core Team, 2019) using ggplot2 3.3.0 (Wickham, 2016) and ggsignif 0.6.0 (Ahlmann-Eltze, 2019).

RTs for responses to target items were analyzed using two LMER models, one fit to all words, and one fit to complex words only, to determine a possible effect of transition probability. Before analysis, RTs were trimmed to discard responses less than 300 ms or more than 1,000 ms from stimulus onset, and RT was log transformed. A graphical summary of RT is shown in Figure 5.

Fixed effects included in the full model were: *condition* (morphologically simple, circumfixed, pseudo-infixed, infixed, [+i] nontransparent pseudoreduplicated, [−i] transparent pseudoreduplicated, reduplicated), log-transformed *item frequency*, and item *length* in letters. After reducing from a maximal model, random intercepts for participant and item were also included in the model, as well as a by-subject slope for *item frequency*. GVIF was calculated to check for collinearity, with no GVIF greater than 1.83. Length was correlated with response speed (*t*(259) = 6.81, *p* < 0.001); longer words were responded to more slowly than shorter words. Frequency was also correlated (*t*(88) = −4.33, *p* < .001), with more frequent words being recognized more quickly.

Treatment coding was specified, allowing for a comparison of conditions to the morphologically simple condition. Two of the morphologically complex conditions were significantly different from the morphologically simple condition when controlling for length and frequency (reduplicate *t*(247) = 2.16, *p* = 0.032; infix *t*(239) = 3.61, *p* < 0.001). However, despite predictions from the MEG results supporting the automatic decomposition of pseudo-infixed words, there was no significant difference between pseudo-infixed and morphologically simple words (*t*(224) = −1.00, *p* = 0.32). The MEG results also supported automatic decomposition for [+i] nontransparent pseudoreduplicated words. For the behavioral results, the difference between [+i] words and morphologically simple words was not significant (*t*(254) = 1.80, *p* = 0.07). On the other hand, the MEG results do not support the automatic decomposition of [−i] transparent words. In this, the behavioral results agree, since those results are not significant either (*t*(241) = 0.279, *p* = 0.78). A summary of the model is shown in Table 5.

**Table 5.** Summary of LMER showing correlation coefficients of RT, lexical statistics, and word types to RT

**All words:**				
**Formula:** RTlog ∼ Condition + Freqlog + Length + (1 | Subject) + (1 | Item) + (WordFreq|Subject)				
**Fixed effects**:	**Estimate**	***df***	***t* value**	**Pr(>|*t*|)**
(Intercept)	−8.66	175	2,66.918	<2e−16***
Condition = circumfix	0	250	0.054	0.957
Condition = pseudo-infix	−0.02	224	−0.996	0.32
Condition = infix	0.06	238	3.614	0.000368***
Condition = pseudoredup [+i]	0.05	254	1.800	0.0731
Condition = pseudoredup [−i]	0.01	241	0.279	0.7807
Condition = reduplicate	0.05	247	2.163	0.0315*
Length	0.03	259	6.805	6.99e−11***
Word Frequency	−0.02	88	−4.331	3.94e−05***
Signif. codes: 0 ‘***’ 0.001 ‘**’ 0.01 ‘*’ 0.05 ‘.’ 0.1 ‘ ’ 1				
**Random effects:**	**Variance**	**Correlation**		
Subject	3.829e−03			
Word Frequency|Subject	9.685e−05	0.54		
Item	4.758e−03			
Residual	2.366e−02			

*Note*. Treatment coding is specified, allowing for a comparison of conditions to the morphologically simple condition. Estimates have been rounded to 2 decimal places. Calculation of *p* values from *t* tests and *df*s was performed using Satterthwaite’s method in the lmerTest package (Kuznetsova et al., 2017).

#### Accuracy

Overall, accuracy rates were high for both blocks, with an average of 91% accuracy across subjects and items. A binomial logit generalized linear mixed-effects model was fit to analyze accuracy, using *log RT* as a predictor (following Davidson & Martin, 2013). In addition to RT, item *condition*, *log frequency*, and item *length* were included in the model. Inclusion of random slopes and intercepts was reduced iteratively starting from a maximal model as described above, resulting in a model with by-subject and by-item intercepts. GVIF was calculated to check for collinearity, and no GVIF was found to be greater than 1.90.

Frequency was found to be a significant predictor of accuracy (*z* = 2.72, *p* = 0.00646). As shown in Table 6, simple words were set as the reference level with treatment coding for levels of condition. Reduplicated words were found to be significantly different from simple words (*z* = 2.32, *p* = 0.02044). The summary of the full model is shown in Table 6.

**Table 6.** Summary of binomial mixed effect logistic regression showing correlation coefficients of RT, lexical statistics, and word types to accuracy

**All words:**			
**Formula:** Accuracy ∼ Condition + RTlog + Freqlog + Length + (1 | Subject) + (1|Item)			
**Fixed effects:**	**Estimate**	***z* value**	**Pr(>|*z*|)**
(Intercept)	−0.40	−0.08	0.93754
Condition = circumfix	1.13	1.44	0.14974
Condition = pseudo-infix	0.81	1.43	0.15234
Condition = infix	0.98	1.81	0.07059
Condition = pseudoredup [+i]	−0.50	−0.67	0.50561
Condition = pseudoredup [−i]	−0.65	−0.92	0.35801
Condition = reduplicate	1.66	2.32	0.02044*
log(RT)	0.60	1.02	0.30701
Length	−0.19	−1.19	0.23285
log(Frequency)	0.30	2.72	0.00646**
Signif. codes: 0 ‘***’ 0.001 ‘**’ 0.01 ‘*’ 0.05 ‘.’ 0.1 ‘ ’ 1			
**Random effects:**	**Variance**		
Subject	0.9919		
Item	2.4244		

*Note*. Treatment coding is specified, allowing for a comparison of conditions to the morphologically simple condition.

## DISCUSSION

As outlined in detail in the introduction, the present study focused on three questions: Are reduplication, circumfixation, and infixation subject to automatic decomposition by the visual system? Furthermore, are words that superficially appear to be reduplicated or infixed but that lack the morphosyntactic and semantic features of such words treated as complex words by the visual system? Finally, is the tendency for a word to be treated by the visual system like a reduplicated word modulated by its conformity to phonological rules?

We addressed these questions by measuring activity elicited in the putative VWFA in anterior fusiform gyrus. The major findings are outlined below. In sum, results from the present study are largely consistent with theories of visual word processing that incorporate automatic decomposition of a word into its stem and affixes (Crepaldi et al., 2010; Taft, 1979, 2004; Taft & Forster, 1975). The present study makes two novel contributions to the literature concerning this topic: First, it adds typological breadth through the inclusion of the understudied language Tagalog, and second, it demonstrates that words formed via previously unstudied morphological processes are also decomposed during visual word recognition. Furthermore, the current study presents further evidence, previously attested for the English irregular past tense (Fruchter et al., 2013), of a mechanism for early automatic decomposition at the intersection of morphology and phonology: For a pseudo-complex word, if phonological rules analogous to those for a complex word seem applicable, the pseudo-complex word will be automatically decomposed, despite the lack of any morphosyntactic indicators of complexity. However, our current results diverge from previously attested constraints of morphosyntactic congruency or stem viability as pseudo-infixed words appear also to be automatically decomposed despite a lack of stem viability without the affix.

### Automatic Early Decomposition of Infixed, Reduplicated, and Circumfixed Words

Segmental information is used by the early visual system to decompose many types of complex words, including those formed by some process other than affixation, namely reduplication. This is evidenced by the effect of stem:whole word transition probability on elicited activity in the left hemisphere. These results are consistent with a robust collection of results from previous studies on suffixation in English (Gwilliams & Marantz, 2018; Lewis et al., 2011; Solomyak & Marantz, 2010) and Greek (Neophytou et al., 2018). Furthermore, Stockall et al. (2019), determined that early automatic form-based decomposition of prefixed English words followed a similar pattern to suffixed words, differing only in hemisphere laterality.

The results of the current study with respect to activity in the left-hemisphere VWFA for morphologically complex words are also noteworthy because of the significant interaction between stem:whole word transition probability and word type. Reduplicated words elicit greater activity for higher values of stem:whole word, which is consistent with both the prefix and suffix literature (Table 1). However, infixed words exhibit the opposite pattern. It is possible also that a single stem:whole word transition probability value for infixed words is not sufficient to completely capture their morphological structure, as they have two morpheme boundaries where the infix meets the stem at both its left and right edges. What remains true, despite the direction of the correlation between transition probability and dSPM, is that transition probability for all complex words correlated with activity in left VWFA.

### Decomposition of Words with Orthophonemic Strings That Imitate Infixes

Our results in support of the automatic decomposition of words with pseudo-infixes diverge from results of previous studies on English, which have investigated underlying rules governing visual morpheme representations. Three different kinds of pseudo-complex items have been investigated in English: words like *brother*, which contain a viable free stem *broth* as well as the viable affix -*er*; words like *winter*, which have the affix, but no viable stem; and words like *vulnerable*, which similarly have no viable free stem, but differ from *winter*-type words in that the affix makes the same contribution to the syntax and semantics of the whole word as it does in clearly complex words like *workable*. The suffix -*ble* creates adjectives with “possibility” semantics (Oltra-Massuet, 2013) in both *workable* and *vulnerable* (compare *winter*, which is neither an agentive nominal nor a comparative adjective).

Tagalog pseudo-infixed words are most similar to English *winter*-type words: Removing the infix does not leave a viable stem, and the whole word does not have the grammar that would be expected if it contained the infix -*in*-. Despite this, we presented results consistent with the hypothesis that pseudo-infixed words are automatically decomposed anyway: Values of activity from both pseudo-infixed and infixed words were compared using a Bayesian estimation, indicating that the values were probably very similar. However, the behavioral evidence did not show that pseudo-infixed words were processed at a different speed than other morphologically simple words; truly morphologically infixed words were.

### Morphologically Simple Pseudoreduplicated Words Imitate Morphologically Complex Reduplicated Words in Their Application of Phonological Rules

The current study compared two types of pseudoreduplicates: those that imitated truly complex reduplicated words in their phonology ([+i]; non-transparent) and those that applied phonological rules as expected for morphologically simple words ([−i]; transparent). The former elicited activity patterns consistent with automatic decomposition as if they were morphologically complex, whereas the latter did not. Therefore, conformity to phonological rules modulates the decomposability of pseudoreduplicated words.

Morphophonological generalizability aiding in the segmentation of complex and pseudo-complex words follows from previous research on English irregular past tense processing. Fruchter et al. (2013) demonstrated that irregular verbs are decomposed into stems and affixes in early written word recognition by correlating priming within the M170 time window to an irregular verb’s conformity to a morphophonological rule (formalized computationally by Albright & Hayes, 2003).

### Conclusion

Our results make several important contributions to our understanding of the neural correlates of morphological decomposition. First, reduplication, infixation, and circumfixation are all comparable to prefixation and suffixation in that they are automatically parsed by the ventral visual system during word recognition, as evidenced by stem:whole word transition probability correlations with activity in VWFA. Additionally, we posit that phono-orthographic cues to morpheme boundaries aid in this automatic decomposition process, as words that are not reduplicated but appear to be so superficially due to their under- and over-application of phonological rules are also decomposed. Collectively, these results are consistent with models of visual word recognition that entail automatic decomposition for all morphological processes.

## ACKNOWLEDGMENTS

The authors would like to extend sincere thanks to Jianjun Hua for statistical consultation, to M. Julieta Guzman and Nathan Quimpo for their assistance in stimuli creation, and to the members of the New York University and New York University Abu Dhabi Neuroscience of Language Lab.

## FUNDING INFORMATION

Alec Marantz, New York University abu Dhabi (https://dx.doi.org/10.13039/100012025), Award ID: Institute G1001.

## AUTHOR CONTRIBUTIONS

**Samantha Wray**: Conceptualization: Supporting; Data curation: Lead; Formal analysis: Equal; Investigation: Lead; Methodology: Supporting; Software: Lead; Validation: Lead; Visualization: Lead; Writing – original draft: Lead; Writing – review & editing: Equal. **Linnaea Stockall**: Conceptualization: Equal; Formal analysis: Equal; Methodology: Equal; Supervision: Equal; Validation: Supporting; Visualization: Supporting; Writing – original draft: Supporting; Writing – review & editing: Equal. **Alec Marantz**: Conceptualization: Equal; Formal analysis: Equal; Funding acquisition: Lead; Methodology: Equal; Resources: Lead; Supervision: Equal; Validation: Supporting; Writing – original draft: Supporting; Writing – review & editing: Equal.

## TECHNICAL TERMS

Morphemes: The minimal units of words that have meaning.
Transition probability: The likelihood of one unit occurring with another, such as a word and the stem it contains.
Phonological: Concerned with the grammatical system governing perceptually unique units of sound in a spoken language.
Phonological transparency: The relationship between a phonological process and the surface phonological form.
