# Supplementary material for: Early Form-Based Morphological Decomposition in Tagalog: MEG Evidence from Reduplication, Infixation, and Circumfixation
Source: Neurobiol Lang (Camb). 2022 Feb 16;3(2):235–55. doi: 10.1162/nol_a_00062 (PMC10158618; doi:10.1162/nol_a_00062)
Supplement: Supplementary file 1 [file nol-3-2-235-s001.docx]

Supplementary Material A: Right-hemisphere analysis

Formula: dSPM ~ base_dSPM + TP * condition + Length + Freqlog + (1 | Subject)

Fixed effects:

|  | Estimate | df | t value | Pr(>\|t\|) |
| --- | --- | --- | --- | --- |
| (Intercept) | -0.20 | 519.6 | -0.503 | 0.615 |
| Base dSPM | -0.14 | 4332 | -9.383 | 2e-16 *** |
| Transition Probability | 1.27e-03 | 4315 | 0.004 | 0.997 |
| Condition = simple | 0.12 | 4315 | 0.405 | 0.685 |
| Condition = pseudo-infixed | -0.17 | 4315 | -0.599 | 0.549 |
| Condition = pseudoredup [+i] | -0.06 | 4315 | -0.197 | 0.844 |
| Condition = pseudoredup [-i] | 0.26 | 4315 | 0.828 | 0.408 |
| Condition = circumfix | -0.21 | 4315 | -0.968 | 0.333 |
| Condition = infixed | -0.12 | 4315 | -0.449 | 0.653 |
| Length | 0.05 | 4315 | 1.374 | 0.169 |
| Base Frequency | -8.79e-04 | 4315 | -0.031 | 0.975 |
| Interaction, TP:Condition = circumfix | 0.21 | 4315 | 0.473 | 0.636 |
| Interaction, TP:Condition= infixed | -0.33 | 4315 | -0.765 | 0.444 |

Signif. codes: 0 ‘***’ 0.001 ‘**’ 0.01 ‘*’ 0.05 ‘.’ 0.1 ‘ ’ 1

Random effects:

|  | Variance |
| --- | --- |
| Subject | 0.5413 |
| Residual | 6.7971 |

Table 7: Summary of LMER showing correlation coefficients of lexical statistics and word types to source component amplitudes (*right* hemisphere). Treatment coding was used for the categorical predictor condition, with the *reduplicate* condition serving as the reference level. Estimates have been rounded to 2 decimal places. Calculation of p values from t-tests and dfs was performed using Satterthwaite’s method in the lmerTest package (Kuznetsova, Brockhoff & Christensen 2017).
